# Impact of the Ebola outbreak on *Trypanosoma brucei gambiense* infection medical activities in coastal Guinea, 2014-2015: A retrospective analysis from the Guinean national Human African Trypanosomiasis control program

**DOI:** 10.1371/journal.pntd.0006060

**Published:** 2017-11-13

**Authors:** Mariame Camara, Eric Ouattara, Alexandre Duvignaud, René Migliani, Oumou Camara, Mamadou Leno, Philippe Solano, Bruno Bucheton, Mamadou Camara, Denis Malvy

**Affiliations:** 1 Programme National de Lutte contre la Trypanosomiase Humaine Africaine PNLTHA-Ministère de la Santé, Conakry, République de Guinée; 2 Univ. Bordeaux, Inserm, Infectious Diseases in Resource Limited Countries, U1219, ISPED, Bordeaux, France; 3 Department of Tropical Medicine and Clinical International Health, CHU Bordeaux, Bordeaux, France; 4 Programme PAC-CI/ANRS Research Site, CHU Treichville, Abidjan, Côte d’Ivoire; 5 UMR 177 IRD-CIRAD INTERTRYP, IRD, Montpellier, France; Institute of Tropical Medicine, BELGIUM

## Abstract

**Background:**

The 2014–2015 Ebola outbreak massively hit Guinea. The coastal districts of Boffa, Dubreka and Forecariah, three major foci of Human African Trypanosomiasis (HAT), were particularly affected. We aimed to assess the impact of this epidemic on sleeping sickness screening and caring activities.

**Methodology/Principal findings:**

We used preexisting data from the Guinean sleeping sickness control program, collected between 2012 and 2015. We described monthly: the number of persons (i) screened actively; (ii) or passively; (iii) treated for HAT; (iv) attending post-treatment follow-up visits. We compared clinical data, treatment characteristics and Disability Adjusted Life-Years (DALYs) before (February 2012 to December 2013) and during (January 2014 to October 2015) the Ebola outbreak period according to available data. Whereas 32,221 persons were actively screened from February 2012 to December 2013, before the official declaration of the first Ebola case in Guinea, no active screening campaigns could be performed during the Ebola outbreak. Following the reinforcement and extension of HAT passive surveillance system early in 2014, the number of persons tested passively by month increased from 7 to 286 between April and September 2014 and then abruptly decreased to 180 until January 2015 and to none after March 2015. 213 patients initiated HAT treatment, 154 (72%) before Ebola and 59 (28%) during the Ebola outbreak. Those initiating HAT therapy during Ebola outbreak were recruited through passive screening and diagnosed at a later stage 2 of the disease (96% vs. 55% before Ebola, p<0.0001). The proportion of patients attending the 3 months and 6 months post-treatment follow-up visits decreased from 44% to 10% (p <0.0001) and from 16% to 3% (p = 0.017) respectively. The DALYs generated before the Ebola outbreak were estimated to 48.7 (46.7–51.5) and increased up to 168.7 (162.7–174.7), 284.9 (277.1–292.8) and 466.3 (455.7–477.0) during Ebola assuming case fatality rates of 2%, 5% and 10% respectively among under-reported HAT cases.

**Conclusions/Significance:**

The 2014–2015 Ebola outbreak deeply impacted HAT screening activities in Guinea. Active screening campaigns were stopped. Passive screening dramatically decreased during the Ebola period, but trends could not be compared with pre-Ebola period (data not available). Few patients were diagnosed with more advanced HAT during the Ebola period and retention rates in follow-up were lowered. The drop in newly diagnosed HAT cases during Ebola epidemic is unlikely due to a fall in HAT incidence. Even if we were unable to demonstrate it directly, it is much more probably the consequence of hampered screening activities and of the fear of the population on subsequent confirmation and linkage to care. Reinforced program monitoring, alternative control strategies and sustainable financial and human resources allocation are mandatory during post Ebola period to reduce HAT burden in Guinea.

## Author summary

This work was conducted in coastal Guinea, the last focus in Western Africa where the transmission of Human African Trypanosomiasis (HAT) is still very active. The Guinean government and his partners are conducting HAT control activities to reduce the burden of this neglected tropical disease and, as set-up by WHO, to eliminate it as a public health problem by 2020. Unfortunately, control efforts were deeply impaired during the Ebola outbreak that stroke the country in 2014–15. The aim of the study was to evaluate the impact of this unprecedented outbreak on HAT screening and caring activities. A major impact was the interruption of all active screening activities which aim, in addition of detecting and treating patients, is to clear the human reservoir of parasite and decrease transmission. Passive surveillance and diagnosis were also severely affected due to the fear of the population to come to the endemic district confirmation and treatment facilities and to the progressive banning of using rapid test in peripheral health structures, as well as the requisition of healthcare workers and facilities for the fight against Ebola. As a consequence, only 59 HAT patients were diagnosed and treated during the Ebola outbreak (from January 2014 to October 2015) as compared to 154 before the outbreak (from February 2012 to December 2013). This potentially high number of undiagnosed human reservoir of trypanosomes may have contributed in turn to increase transmission levels. A rapid revival of HAT control activities in Guinea is thus vital in order to stay in line with the 2020 elimination goal and to avoid possible bursts of the disease.

## Introduction

Human African trypanosomiasis (HAT) is a vector born parasitic disease due to the infection by *T*. *brucei gambiense* in Western and Central Africa and *T*. *brucei rhodesiense* in Eastern Africa. In its late stage, HAT is generally considered to be fatal if left untreated and is responsible for a heavy burden in affected areas. It is one of the most neglected tropical diseases and, until recently, the treatment relied on ancient and toxic drugs [1]. In Western Africa, most cases of *T*. *brucei gambiense* infection are found in remote sparse active focuses such as the three major ones situated in the mangrove ecosystem of coastal Guinea[2], namely from North to South: Boffa [3–4], Dubreka [4] and Forecariah [4–6]. Of note, these three districts were among the most affected by the epidemic of Ebola virus disease that severely hit Guinea in 2014–2015 [7].

Previous studies have demonstrated the impact of the recent Ebola outbreak on health systems of affected countries with an unprecedented magnitude on HIV care or malaria control activities in Guinea and Liberia [8–13]. First, the Ebola crisis has weakened an already fragile healthcare system by the re-allocation of the available resources to fight Ebola. In addition, health-care workers who were in first-line have paid a heavy toll with 1.5% deaths among doctors, nurses, and midwives compared to 0.02% in the general population of Guinea [14]. Second, Ebola burden has led to a drastic reduction in healthcare center attendances [9]. The high transmission rates of Ebola virus in healthcare centers had raised a justifiable fear against the health system in general with dramatic consequences[15,16].

Since HAT control activities are generally vulnerable and poorly supported in terms of human or financial resources, one might expect that the Ebola epidemic has deeply impaired HAT control as well. Therefore, we aimed at investigating the impact of the Ebola outbreak on HAT screening and caring activities in the three coastal Guinean focuses of Boffa, Dubreka and Forecariah.

## Methods

### Settings

The Guinean national HAT control program is devoted to reducing the burden of sleeping sickness through a comprehensive action: (i) fight against the vector with targeted vector control using insecticide impregnated targets; (ii) improving human infection diagnosis by reinforcing passive screening with the implementation of new rapid diagnostic tests at peripheral level (health posts and centers) and conducing targeted active screening campaigns; (iii) treatment and follow-up of patients diagnosed with HAT. Our analysis focuses on screening and caring (HAT diagnosis confirmation and subsequent treatment and follow-up) purposes.

**Fig 1:**
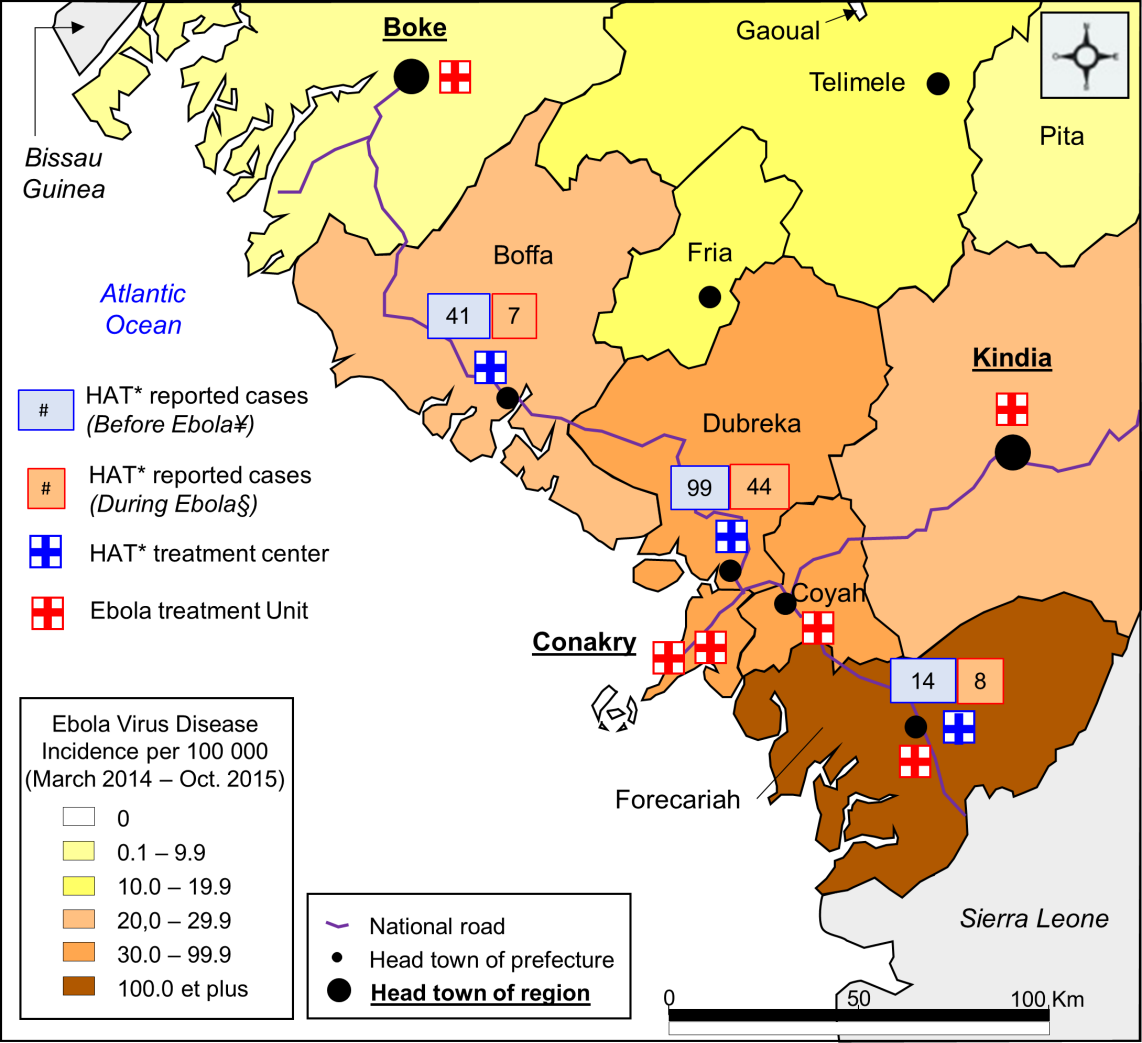
Ebola virus disease and HAT treatment centers spatial distribution. This figure shows the spatial distribution of Ebola virus disease (EVD) incidence with both EVD and Human African Trypanosomiasis (HAT) treatment centers and newly diagnosed HAT cases during the study period (before and during Ebola outbreak) in coastal Guinea. *HAT: Human African Trypanosomiasis. ¥ Before Ebola: from Feb.2012 to Dec.2013. § During Ebola: from Jan.2014 to Oct.2015.

Our study was carried out in three districts of coastal Guinea, namely from north to south Boffa, Dubreka and Forecariah (Fig 1). The Boffa [3,4] and Dubreka [4] districts are located at the north of the capital city Conakry and Forecariah [4–6] district is located at the south of Conakry. All districts are situated in a mangrove ecosystem favoring the growth of the HAT vector population. The area mainly hosts Soussou and Baga ethnic groups. Inhabitants have outside activities such as irrigated rice growth, salt extraction, wood cut, and fishing [3] exposing them to high contacts with tsetse flies. In 2005, the prevalence of HAT in the area was estimated to be about 1% [17]. The number of newly reported HAT cases was 78 in 2013 and increased to 106 cases in 2016[18].

**Fig 2:**
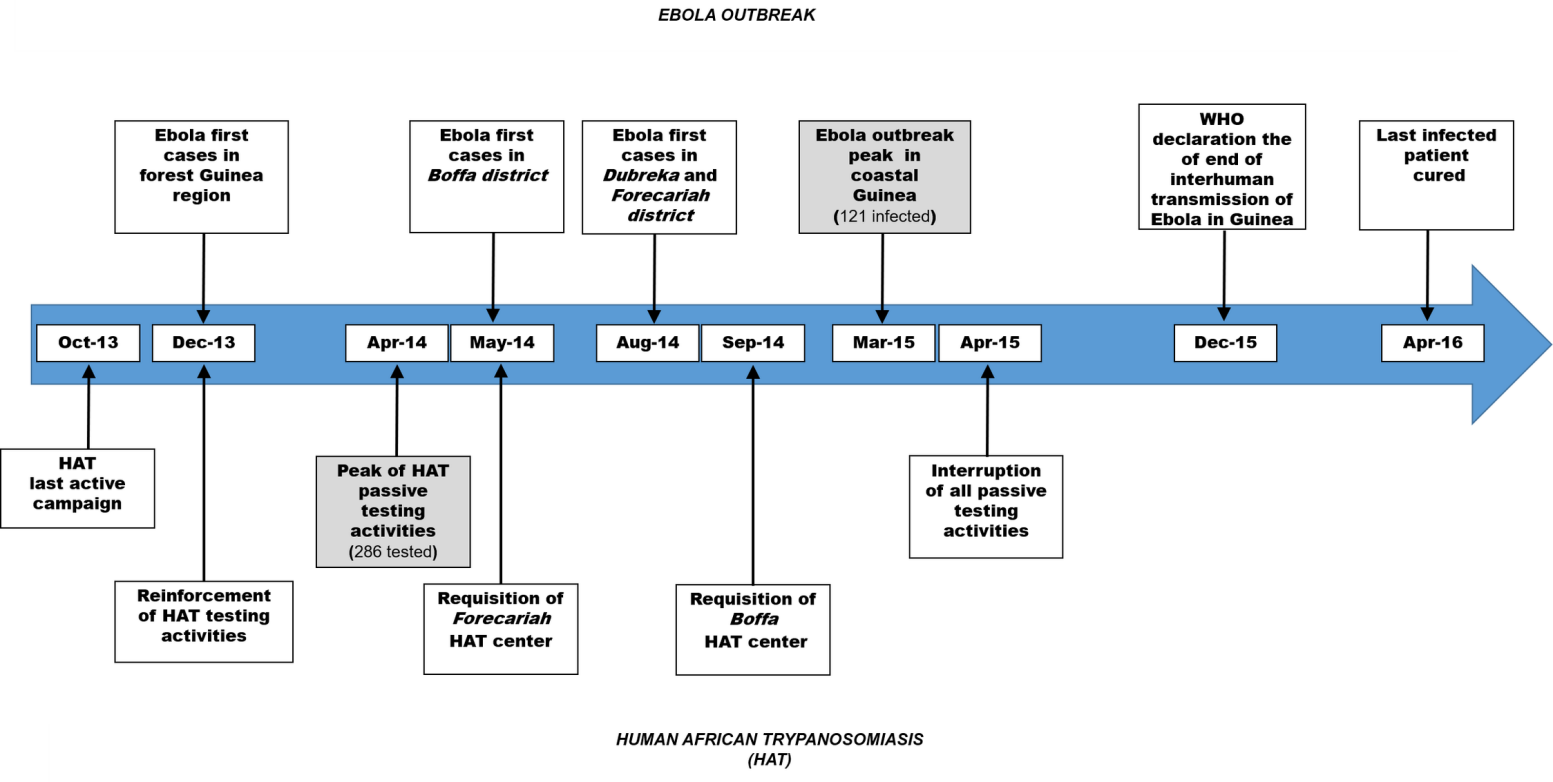
Timeline of the Ebola epidemic and HAT control activities. This figure shows in parallel the main events of the Ebola epidemic and of Human African Trypanosomiasis (HAT) control activities between October 2013 and April 2016.

An unprecedented Ebola outbreak struck Guinea from December 2013 to April 2016 with more than 3,800 persons infected of whom 2,500 died [19]. The epidemic started in South East Guinea region and reached Conakry and coastal Guinea around March 2014. Many healthcare centers including HAT treatment centers were requisitioned for the fight against Ebola (Fig 2).

### Study and population

We performed a retrospective analysis of the data of all the people screened and/or treated for HAT in the three districts of Boffa, Dubreka and Forecariah within the frame of the Guinean HAT National Control Program (PNLTHA) routine activities. For that purpose, we used available data collected between February 2012 and October 2015 in two electronic databases: (i) the ‘HAT treatment database’ collecting prospectively sociodemographic, clinical, parasitological, therapeutic and follow-up information of the patients initiating HAT treatment in the study centers between January 2012 and October 2015; (ii) the ‘HAT screening database’ recording active testing campaigns activities since 2012 and passive screening activities only since 2014. Regarding Ebola, we used previously published data reporting monthly cases in Guinea between March 2014 and October 2015 [19]. Data deposited in the Dryad repository, https://doi.org/10.5061/dryad.036ds [20]. HAT-infected persons were recruited during active screening campaigns throughout the entire districts or by passive detection in persons presenting spontaneously to the three HAT treatment centers based in the Forecariah, Dubreka and Boffa cities and, as from 2014, some people presenting at peripheral health centers of the same districts who underwent decentralized screening before the interdiction of blood sampling due to Ebola. Indeed, early in 2014, HAT passive detection capacities were reinforced by the implementation of a passive surveillance system in which SD-Bioline rapid diagnostic tests [21] were made available at the level of the endemic district’s peripheral health centers (approximately 30 per disease focus). Additional cases were also actively searched by targeted screening of the neighborhood of previous cases. Positive serological suspects were referred to the endemic district laboratories for parasitological study and if confirmed, treatment was performed. Individuals with positive Card Agglutination Test for Trypanosomiasis (CATT) underwent lymph node puncture when relevant and /or blood sampling for direct diagnostic confirmation according to previously described procedures [22]. Cerebrospinal fluid collection by lumbar puncture in confirmed cases allowed disease staging and treatment guidance [22]. Patients with 5 or less white blood cells (WBC)/ml of CSF were considered as stage 1 HAT patients whereas those with 6 or more cells were classified as stage 2 patients. The persons diagnosed with HAT were admitted in HAT treatment centers situated close to the endemic district laboratories. Boffa, Dubreka and Forecariah centers have a capacity of respectively 17 beds, 16 beds and 4 beds. After a treatment preparation phase consisting in pain relief, deworming and administration of an anti-malaria treatment, pentamidine isethionate (4 mg/kg) was administered intramuscularly during 7 days to patients at the stage 1 of the disease. The NECT regimen (nifurtimox at the dosage of 15 mg/kg/day divided in three oral intakes for 10 days and eflornithine 400 mg/kg/day administered in two intravenous infusions for 7 days) was used for patients with stage 2 disease. Post-treatment follow-up visits were planned 3, 6 and 12 months after treatment completion according to national procedures applicable at that time.

### Outcomes and statistical analysis

We evaluated the impact of Ebola outbreak on HAT screening activities by analyzing overtime the number of persons screened during active campaign and the number of persons screened passively during the 2014–2015 period (data were not available for passive screening before 2014).

To evaluate the impact of Ebola outbreak on caring activities, we first analyzed overtime the overall number of patients initiating HAT treatment and the number patients who attended the follow-up visits 3 and 6 months after HAT treatment was completed. Then, we compared sociodemographic, clinical, treatment and follow-up variables before and during Ebola outbreak using Fisher exact tests. The period referred as ‘before Ebola period’ ran from February 2012 (beginning of available data in the “HAT treatment database”) to December 2013 (the first cases of Ebola appeared in late december 2013 in “Guinée forestière”) and the ‘Ebola period’ extended from January 2014 (first plain month after the first Ebola cases in Guinea and beginning of the “HAT screening database”) to October 2015 (end of available data).

We estimated Disability Adjusted Life-Years (DALYs) before and after the Ebola crisis, using the same methodology than the 2010 Global Burden of Disease study, without application of age-weighting and discounting [23]. We weighted average DALYs for the following 3 sub-groups: (i) reported HAT cases before Ebola; (ii) reported cases during Ebola; (iii) under-reported cases during Ebola. The latter group refers to HAT cases that were not reported during the Ebola period compared to what has been observed before the Ebola outbreak, assuming that HAT incidence remained stable during Ebola and the decrease in the number of newly diagnosed HAT cases was attributable mainly to Ebola outbreak. Those assumptions are in line with previous reports from WHO [18]. Reported deaths and life expectancy at the age of death were used to estimate the Years of Life Lost (YLLs) in both sub-groups (i) and (ii). Under-reported deaths were generated from binomial distributions with assumptive case fatality rates of 2%, 5% and 10% corresponding to conservative, average and pessimistic scenarios. Years Lived with Disability (YLDs) were estimated using disability weighting of 0.21 for early disease stage, 0.35 for late advanced stage; treatment duration of 1 month and time to death for untreated patients at 3 years [23]. We computed 95% confidence intervals around estimates using bootstrap (1,000 patients resampling, for detailed methodology see S1 Text).

Statistical analyses were performed using SAS 9.3 (SAS Institute, Cary, North Carolina, USA).

### Ethics

All HAT patients described in the framework of this study were diagnosed and treated according to the national health and WHO policy. Agreement was obtained from the Ministry of Health of Guinea to use the National Control Program database (2012–2016) to assess the impact of Ebola on sleeping sickness. Data were anonymized prior to the analysis. All the investigations were conducted in accordance with the declaration of Helsinki. This work fulfills the STROBE criteria.

## Results

### Active screening campaigns (2012–2015)

**Fig 3:**
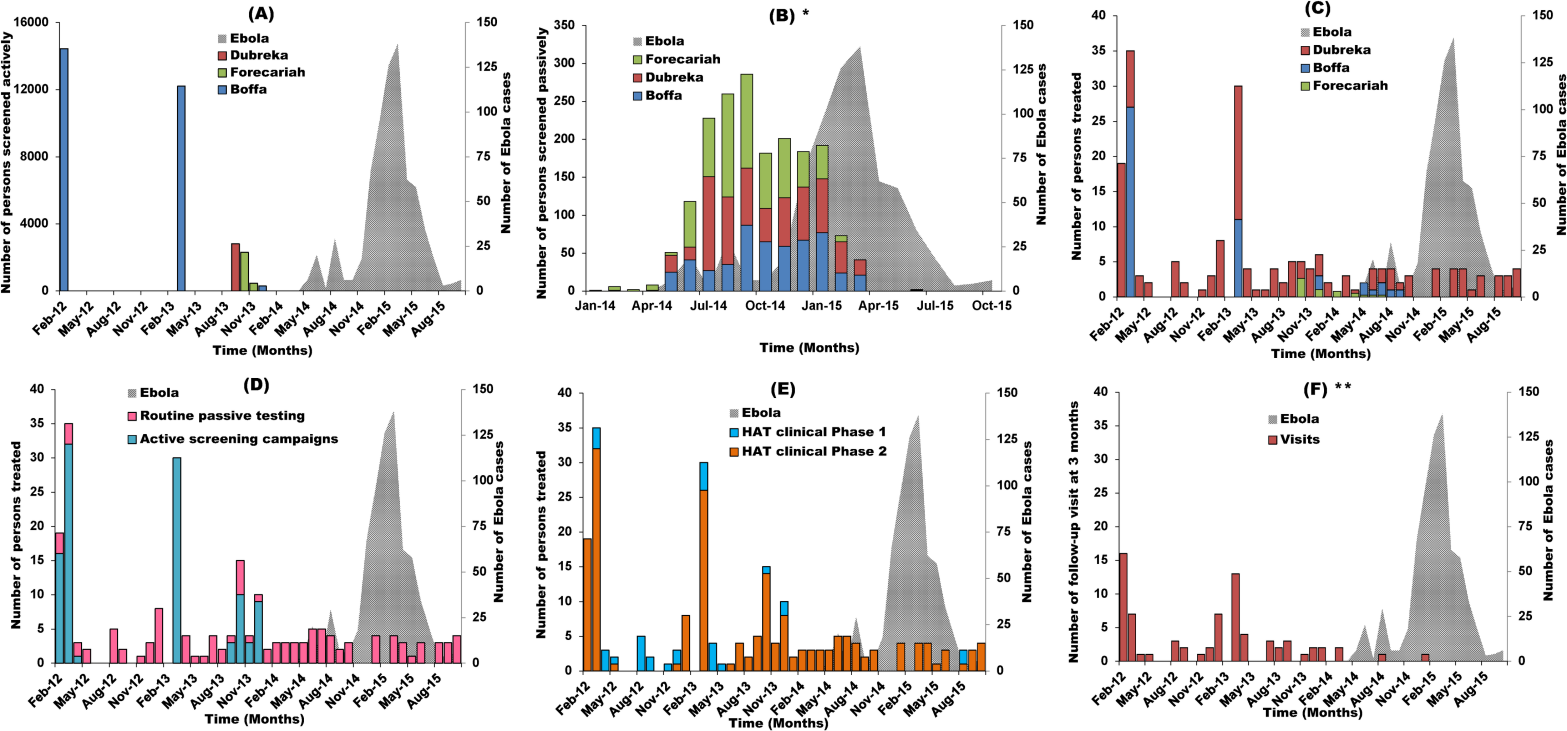
Impact of Ebola outbreak on HAT testing and caring activities in Guinea, January 2012 to October 2015. This panel figure displays: (A) Monthly evolution of the number of persons diagnosed during active campaigns by HAT treatment center; (B) Monthly evolution of the number of persons tested passively by HAT treatment center (and as from 2014 corresponding district); (C) Monthly evolution of the number of persons initiating therapy by HAT treatment center; (D) Monthly evolution of the number of persons initiating HAT therapy according to the type of screening; (E) Monthly evolution of the number of persons initiating HAT therapy by disease stage at diagnosis (F) Monthly evolution of the number of persons attending 3 months post-treatment follow-up visit. HAT: Human African Trypanosomiasis: *Passive routine testing data were available only between January 2014 and October 2015. **All post-treatment follow-up visits were centralized at the Dubreka center whatever the place where the patients received HAT therapy (Dubreka, Boffa or Forecariah HAT centers).

Fig 3A shows the monthly number of persons screened during active campaigns. Overall 32,221 persons were screened for HAT during 6 active campaigns led since February 2012. The last active screening survey was performed in December 2013 during the follow-up of previously treated patients at their home. A total of 291 family members or neighbors were tested for HAT during this activity. No other active screening campaign was led out after that, during the Ebola period.

Among the persons screened in the 2012–2013 period, 26,950 (84%), where from the Boffa district as the result of an elimination project initiated in 2012 in this area[24]. Three lesser campaigns were led in September 2013 with 2,810 persons screened in Dubreka, in October 2013 and November 2013 with respectively 2,299 and 453 persons screened in Forecariah.

### Passive screening activities (2014–2015)

Overall 1,836 persons were tested passively for HAT between January 2014 and October 2015. Fig 3B shows the monthly number of persons tested during this period. Passive testing at peripheral heath centers was performed at a low level in Dubreka and Forecariah districts from January 2014 to April 2014 as the passive surveillance system was reinforced. In Boffa, activities started slightly later in May 2014. After April 2014, the number of persons tested passively rapidly increased to reach a peak of 286 persons in September 2014. After September 2014, the number of tests abruptly decreased to a plateau of about 180 persons until January 2015. From there, the number of tests rapidly decreased to no testing after February 2015 in Forecariah and after March 2015 in Boffa and Dubreka with the interdiction to perform Rapid Detection Testing (RDT) in peripheral health centers. The referral rate of individuals testing positive at a health center was also very low as only 18% (13 of 70 serological suspects) were indeed seen for confirmation at the HAT treatment centers (of whom 7 were confirmed as HAT patients). Unfortunately, we were not able to compare this parameter with pre-Ebola period.

### Treatment and follow-up (2012–2015)

Between February 2012 and October 2015, 213 patients were treated for HAT, 154 (72%) before and 59 (28%) during the Ebola outbreak in coastal Guinea (Fig 1). Of note, all patients with confirmed HAT initiated the treatment. During the whole study period, the mean number of HAT patients treated monthly was 5 (Confidence Interval 95% (CI95%): 3–7). This number was 7 (95%CI: 3–11) before the Ebola period and 3 (95%CI: 2–3) during the Ebola period (S1 Table). The number of patients treated for HAT was the highest in March 2012 and March 2013, with 35 and 30 patients respectively, corresponding to large active screening campaigns led in the Boffa focus (Fig 3C, Table 1).

**Table 1:** Characteristics of the patients treated for HAT before and during Ebola outbreak, Guinea, January 2012 to October 2015 (for sensitivity analysis with different split dates between the two periods see S2 Table and S3 Table).

** **	N	Both periods	Before Ebola outbreak (23 months)	During Ebola outbreak (22 months)	*P value*
**Number of patients initiating the treatment**	213	213 (100%)	154 (72%)	59 (28%)	—
**Gender**	212				0.530
Male		130 (61%)	96 (63%)	34 (58%)	
Female		82 (39%)	57 (37%)	25 (42%)	
**Age (years), n (%)**	212				**0.031**
< 18 yrs		51 (24%)	43 (28%)	8 (14%)	
≥ 18 yrs		161 (76%)	110 (72%)	51 (86%)	
**HAT treatment centers** ^**(1)**^**, n (%)**	213				**0.048**
Boffa		48 (23%)	41 (27%)	7 (12%)	
Dubreka		143 (67%)	99 (64%)	44 (75%)	
Forecariah		22 (10%)	14 (9%)	8 (13%)	
**Occupation** ^**(2)**^**, n (%)**	201				0.874
Outside rural activity		73 (36%)	54 (37%)	19 (35%)	
Outside city activity		13 (**7%**)	9 (6%)	4 (7%)	
Inside activity		115 (57%)	83 (57%)	32 (58%)	
**Type of screening, n (%)**	211				**<0.0001**
Passive screening		107 (51%)	49 (32%)	58 (100%)	
Active screening		104 (49%)	104 (68%)	0 (0%)	
**Disease clinic stage** ^(3)^**, n (%)**	213				**<0.0039**
Phase 1		31 (15%)	29 (19%)	2 (4%)	
Phase 2		182 (85%)	125 (81%)	57 (96%)	
**Treatment Status, n (%)**	213				**0.0129**
Treatment completed		179 (96%)	136 (88%)	43 (73%)	
Treatment not completed ^(4)^		32 (15%)	17(11%)	15 (25%)	
Dead		2 (1%)	1 (1%)	1 (2%)	
**Follow-up at 3 months, n (%)**	213				**<0.0001**
No		139 (65%)	86 (56%)	53 (90%)	
Yes		74 (35%)	68 (44%)	6 (10%)	
**Follow-up at 6 months, n (%)**	213				**0.017**
No		187 (88%)	130 (84%)	57 (97%)	
Yes		26 (12%)	24 (16%)	2 (3%)	

HAT: Human African Trypanosomiasis

^(1)^ HAT treatment centers were health centers specialized in the care of HAT

^(2)^ Outside rural activity (farmers, woodcutters, and fishermen); Outside city activity include (taxi drivers, mechanics, house builders, black-smith, and carpenters), inside activity (student, office employees, plumbers, tailors, and photographs)

^(3)^ Patients with 5 or less white blood cells (WBC)/ml of CSF were considered as stage 1 HAT patients whereas those with 6 or more white blood cells (WBC)/ml of CSF were classified as stage 2 HAT patients

^(4)^ Treatment not completed include 4 patients (3 before, 1 during Ebola) transferred to other centers, 2 patients (both before Ebola) who left the healthcare centers without medical authorization and 26 patients with missing data.

Among these patients, 104 (49%) were recruited during active campaigns and 107 (51%) through passive screening (Fig 3D, Table 1). All the patients initiating the treatment during Ebola period were recruited passively (100% vs 32% before Ebola, p<0.0001) (Fig 3D, Table 1).

Regarding the activities of the different treatment centers, 143 (67%), 48 (23%), and 22 (10%) treatments were initiated at Dubreka, Boffa and Forecariah, respectively. The proportion of patient treated in the Dubreka center significantly increased during the Ebola period (75% vs 64% before Ebola, p = 0.048) (Fig 1, Fig 3C, Table 1) as the Forecariah and Boffa centers were requisitioned in May and September 2014 respectively. Overall, 130 patients (61%) were male and 161 (76%) were >18 years old. Of 201 patients with available data, 115 (57%) had inside occupational activities in mangrove area. During EVD period, significantly less patients aged < 18 years old initiated treatment (14% vs 28% before EVD, p = 0.031) (Table 1).

During the Ebola period, significantly more patients were diagnosed with stage 2 disease compared to the pre-Ebola period (96% vs. 81%, p = 0.0039, Table 1). If we consider missing values (n = 26) as failure to complete HAT therapy, more patients did not complete their treatment during Ebola than before (25% vs. 11%, p = 0.0129). Of note, the 2 (1%) patients who died during the study period were admitted in a comatose state and passed before NECT could be initiated. The proportion of patients attending the 3 months and 6 months post-treatment follow-up visits (centralized at the Dubreka center) decreased from 44% to 10% (p<0.0001) and from 16% to 3% (p = 0.017) respectively, before and during Ebola period.

To account for uncertainty concerning the split date chosen (January 2014) between pre-Ebola and Ebola periods, we performed a sensitivity analysis with two different split dates (December 2013 and March 2014). The results remained similar (see S2 Table and S3 Table).

### Disability Adjusted Life Years (DALYs) estimates

Only 1 death was reported for both periods, i.e.; 1 (1%) and 1 (2%) before and during Ebola respectively (Table 1). The 2 patients who died were admitted in a comatose presentation and passed out before the therapeutic regimen could be administered. Hence, assuming a case fatality rate (CFR) of 2% (similar to what has been observed among HAT cases reported during the Ebola period) among the under-reported cases (cases that should have been reported based on previous reports), the estimated number of deaths increased from 1 before Ebola to 3 during Ebola (Table 2). In alternative scenarios based on CFR of 5% and 10%, the number of deaths during Ebola increased to 6 and 10 respectively. YLLs was 44.7 (42.0–47.5) before Ebola and increased after Ebola up to 116.1 (110.8–121.4), 232.2 (225.0–239.7) and 413.7 (403.5–423.9) for under-reported CFR of 2%, 5% and 10% respectively. The YLD estimate increased from 4.0 before Ebola to 101.4 during Ebola for all scenarios. Finally, the DALYs were estimated to 48.7 (46.7–51.5) before Ebola and increased to 168.7 (162.7–174.7), 284.9 (277.1–292.8) and 466.3 (455.7–477.0) during Ebola assuming a CFR in under-reported HAT cases of 2%, 5% and 10% respectively (Table 2, for detailed estimations see S4 Table).

**Table 2:** Disability-Adjusted Life Years (DALY) estimates for HAT cases before and during Ebola outbreak, Guinea (January 2012 to October 2015).

	Before Ebola outbreak (n = 154 reported cases)	During Ebola outbreak (n = 59 reported and 95 under-reported cases) ^(1)^		
		*Conservative scenario (Under-reported CFR = 2%)*	*Average scenario (Under-reported CFR = 5%)*	*Pessimistic scenario (Under-reported CFR = 10%)*
Number of deaths ^(2^^)^	1.0	3.0	6.0	10.0
Years Lost due to Disability (YLD) ^(3)^	4.0	101.4	101.4	101.4
Years of Life Lost (YLL) ^(4)^	44.7 (42.0–47.5)	116.1 (110.8–121.4)	232.2 (225.0–239.7)	413.7 (403.5–423.9)
Disability-Adjusted Life Year (DALY) ^(4)^	48.7 (46.7–51.5)	217.5 (212.2–222.7)	333.6 (326.3–340.9)	515.1 (504.9–525.2)
**Overall DALY increase** **(***During Ebola—Before Ebola***)**	-	**168.7 (162.7–174.7)**	**284.9 (277.1–292.8)**	**466.3 (455.7–477.0)**

CFR: Case Fatality Rate

^(1)^ The number of HAT cases reported was 154 and 59 before and during Ebola respectively. The number of reported cases over the 23 months before Ebola period was consistent with the previous WHO reports [18]. We then assumed that 95 HAT cases, the difference between the cases reported before and during Ebola were under-reported during Ebola period.

^(2)^ The number of deaths in under-reported HAT cases was generated from a binomial distribution. The presented results are rounded.

^(3)^ The 95% confidence intervals are not shown because of very small variations

^(4)^ Values are mean values (with lower and upper 95% confidence intervals) from 1,000 bootstrap simulations

## Discussion

Our study suggests a major impact of the Ebola outbreak on HAT screening activities in the Western Africa most active focuses of Boffa, Dubreka and Forecariah in costal Guinea. All active screening campaigns had to be abandoned during the Ebola outbreak and passive screening activities were also impacted despite new efforts to avail rapid diagnostic tests in peripheral health centers in active foci. The number of person screened passively at health centers was first reduced by half during the early phase of the Ebola outbreak to zero after the outbreak peak. Nevertheless, some patients kept coming by themselves to the only remaining functional treatment center in Dubreka throughout the Ebola period. Even if we were not able to demonstrate a direct impact of Ebola on the referral rate of serological suspects (people with a positive screening test) to HAT centers for diagnostic confirmation and subsequent treatment, the number of patients initiating HAT therapy was reduced by 2/3 during Ebola, with 96% of them diagnosed at a late stage of the disease and a poor post-treatment retention in follow-up. It is unlikely that this drop could be explained only by a fall in HAT incidence. Hence, Ebola outbreak contributed to worsening the burden of HAT in Guinea, given DALYs were increasing from 48.7 to 217.5 in our conservative scenario. This increase in DALYs might have reached up to 466 in the most pessimistic scenario assuming a CFR of 10% among under-reported HAT cases during Ebola outbreak, in line with previous data from WHO [25].

Active screening campaigns, aimed at reducing the human reservoir of trypanosomes are very important for the control of HAT in the hardly reachable mangrove ecosystem of costal Guinea where access to healthcare is limited. The mass screening campaign planned in the Boffa focus in March 2014 had to be abandoned during its awareness phase after the first Ebola cases have been officially declared in Guinea. Prior to the Ebola period, such campaigns provided 68% of the patients treated for HAT and enabled the detection/treatment of persons in the early disease stage 1, when no or only none-specific symptoms are present. Targeting these individuals is of up-most importance as, remaining active, they represent a major source of infection for the vector [26], and because the treatment outcome is by far much favorable. Initially, for evident safety reasons and to avoid population gatherings, and then because of population mistrust toward the health system, all active screening activities had to be abandoned throughout the Ebola period, thereby hindering the effort to reduce the *T*. *brucei gambiense* reservoir and global HAT burden through early diagnosis and treatment. Only 2 (4%) patients were diagnosed in stage 1 during the Ebola period as compared to 29 (19%) before Ebola (Table 1). This sharp deficit highlights the fact that an important number of tsetse infective individuals were left undiagnosed during the Ebola outbreak. It is yet too early to assess precisely the impact of this on *T*. *brucei gambiense* transmission levels, but it is noteworthy that 79 HAT patients were recently diagnosed in the one and only Boffa focus where active screening activities could recently be implemented again (May and October 2016). This sharp increase of HAT prevalence, reaching up to 5% in several villages of the focus, is probably largely attributable to the three-year absence of active screening in these areas (data from Guinean national HAT control plan, to be published later).

With the inability to perform active screening, all medical activities to control HAT in costal Guinea during the Ebola period had to rely on the passive detection of patients. Although the data on the number of persons passively tested for HAT in the three endemic focuses were scarcely available before 2014, passive cases (self-presenting at HAT treatment centers) represented 32% of HAT patients in the pre-Ebola period (Table 1). Data on passive screening were computerized and systematically collected after 2014 when the capacities of Boffa, Dubreka and Forecariah health districts were reinforced by the settlement of a passive surveillance system in which serological suspicion was made at the level of local health centers (# 30 per disease focus) and parasitological confirmation was done at the district treatment centers. The scale-up of reinforced passive screening activities has unfortunately coincided with the Ebola outbreak resulting in a reduction by half in the number of persons tested during the early epidemic. This can be partly explained by the fear of the population to attend health structures, leading to a sharp attendance decrease. In this regard, it is noteworthy that less than 20% (13/70) of the persons who tested positive to the HAT rapid test at the peripheral health center level indeed showed up at one of the districts confirmation laboratories. This phenomenon was amplified by the fact that the HAT confirmation/treatment centers of Forecariah and Boffa were requisitioned early during the Ebola outbreak leaving Dubreka as the only functional center. Hence the mean distance to seek for HAT confirmation and treatment was increased and discouraged many clinical suspects. The use of rapid tests in peripheral health centers was then progressively abandoned with the interdiction of taking capillary blood samples in these areas.

Interestingly, significantly less people aged < 18 years were diagnosed with HAT during Ebola (14% vs. 28%, p = 0.031). Maybe parents were less prone to let their children go outside (or to accompany them to health centers) during this period.

Although late stage diagnosis is a classical feature of HAT passive diagnosis [4], the proportion of patients diagnosed through passive screening with stage 2 disease has increased sharply from 57 to 97% during the Ebola period (S5 Table). This illustrates another indirect impact of Ebola on HAT which was to increase the time length between infection and diagnosis in passively screened patients. Even with the well tolerated NECT regimen, the treatment of advanced infection remains hazardous [27]: greater efforts are required to raise the patient’s health status before initiating anti-trypanosome therapy; advanced stage patients require a constant attention from the medical teams and they often need to stay under observation for several days/weeks after treatment completion before they can be discharged. HAT diagnosis and treatment are provided freely in Guinea. Nevertheless, increase of hospitalization length can represent a heavy economic burden for affected families as food is not supplied and the patients need guardians to look after them. Economic losses are particularly high when the patient’s homes are located far away from the treatment centers. This was the case for many patients during the Ebola period as Dubreka became quickly the only available HAT treatment center in Guinea. Another impact of the fear of Ebola among communities on the patient care is that the proportion of patients coming for follow-up visits after treatment was significantly reduced, even if the new WHO 2014 guidelines which were no longer recommending systematic follow-up visits for HAT treated patients had not yet been implemented in Guinea.

Assessing the impact of Ebola on HAT public health outcomes was not possible with the data available. However, it is noteworthy that no difference was observed in the lethality for the reported cases denoting the tremendous efforts of the medical staff to maintain acceptable treatment condition despite facing major challenges. DALYs estimates using the available data have shown a significant impact of Ebola. Even if these estimates rely on many assumptions due to the lack of evidence, the impact of Ebola outbreak was already substantial with the conservative hypothesis of a similar 2% CFR for reported and under-reported HAT cases during Ebola. Few studies have estimated DALYs for HAT infection, mainly addressing *T*. *brucei rhodesiense* sleeping sickness [28,29]. We however, used comparable DALYs estimation methods and were more conservative about the CFR in under-reported cases in accordance with the lower morbidity of *T*. *brucei gambiense* sleeping sickness [28–30].

Our study has several limitations. First, we used distinct databases with different periods of data collection for active screening, passive screening and treatment. Thus, we were not able to statistically compare the trends in screening activities variation between periods, especially for passive screening. The result of passive screening activities should be interpreted with caution since the improvement of data collection may have induced reporting bias. In addition, results on screening activities before Ebola indirectly derived from the data of the patients treated for HAT may be subject to selection bias. Second, we have no data on adverse events during HAT treatment or post treatment sequelae which combined with mortality may have better captured treatment outcome. Third, our analysis was limited to before and during Ebola periods since the data for post Ebola period were not fully available. Hence, we certainly not have captured all the burden due to HAT cases left undiagnosed, since *T*. *brucei gambiense* HAT is a medium-long term evolution disease. But the DALYs estimation is a way of appreciating this burden despite this limit (even if subject to approximations). Post-Ebola data should comfort our findings by showing expected increasing HAT transmission after Ebola epidemic (preliminary data from Guinean national plan against HAT, to be published later).

Our findings are consistent with previous studies on the impact of Ebola on HIV care in forest Guinea and Liberia [9,11] and indicate an important impact also on HAT control and patient care in costal Guinea. Importantly, it is very likely that the deficit of active screening campaigns and the progressive failure of HAT passive detection have left many undiagnosed *T*. *brucei gambiense* infected persons in endemic areas. This may have created in turn favorable epidemiological conditions for disease burden enhancement in some areas. Increased awareness efforts toward HAT endemic communities as well as the revival of the passive surveillance system together with large active screening campaigns allowing early HAT diagnosis and treatment are thus crucial and timely to more fully evaluate the impact of Ebola on HAT transmission and avoid possible dramatic bursts of disease prevalence in endemic foci. Targeted vector control measures, as those implemented previously in part of the Boffa focus [24] may also help speeding–up the reduction of transmission levels and stay in line with the 2020 elimination goal [31].

Finally, to limit the consequences of such a crisis in the future and make HAT control programs more resilient, it appears crucial that “less vulnerable” strategies such as vector control be generalized and reinforced, alongside with an ambitious and sustainable (in terms of human and financial resources allocation) active screening campaigns program, in order to reduce both transmission and human reservoir. As for Ebola, the involvement of community leaders could probably be a good way of increasing both awareness and adhesion of the population, as well as limiting the costs, thereby improving the efficiency of the entire program.

## Supporting information

S1 Text Disability Adjusted Life-Years (DALY) calculation. (DOCX)

S1 Table Mean number of HAT patients treated before and during Ebola outbreak, Guinea (January 2012 to October 2015). (DOCX)

S2 Table Characteristics of patients treated for HAT before and during Ebola outbreak, Guinea (January 2012 to October 2015), *Cutoff December 2013*. (DOCX)

S3 Table Characteristics of patients treated for HAT before and during Ebola outbreak, Guinea (June 2012 to October 2015), *Cutoff March 2014*. (DOCX)

S4 Table Disability-Adjusted Life Years (DALY) estimates before and during Ebola outbreak, Conakry Guinea 2012–2015 (Extended results). (DOCX)

S5 Table HAT disease clinic stage before and during Ebola outbreak in patients detected through passive testing, Guinea (2012 to 2015). (DOCX)
