# Supplementary material for: Impact of the Ebola outbreak on Trypanosoma brucei gambiense infection medical activities in coastal Guinea, 2014-2015: A retrospective analysis from the Guinean national Human African Trypanosomiasis control program
Source: PLoS Negl Trop Dis. 2017 Nov 13;11(11):e0006060. doi: 10.1371/journal.pntd.0006060 (PMC5703571; doi:10.1371/journal.pntd.0006060)
Supplement: S1 Text — (DOCX) [file pntd.0006060.s001.docx]

**S1 Text. Disability-Adjusted Life Years (DALY) calculation**

DALY calculations followed the general methods used in the 2010 Global Burden of Disease study without age-weighting and discounting ^[1]^. DALY is a disease burden measure combining morbidity and mortality by summing Years of Life Lost (YLL) due to premature mortality and Years Lost due to Disability (YLD) accounting for health condition or its consequences.

1. YLL= (Number of deaths) * (life expectancy at the age of death)
2. YLD = (Number of cases) * (duration till remission or death) * (disability weight)
3. DALY = YLL+ YLD

First, individual YLL and YLD were used to estimate average DALYs for the following sub-groups: (1) reported HAT cases before Ebola (cases that were diagnosed and treated); (2) reported cases during Ebola; (3) under-reported cases during Ebola. Since the number of case reported to WHO was almost constant over the previous years and they were no changes in HAT control activities in Guinea, we assumed that under-reported cases were due to Ebola outbreak. Individual YLLs were calculated using reported deaths in subgroups (1) and (2). For the under-reported cases, Individual YLLs were calculated using deaths generated from Bernoulli distribution with the following case fatally rates: 2%, 5% and 10%, corresponding to optimistic, average and pessimistic scenarios ^[2]^. We used a disability weight of 0.21 for early stage and 0.35 for late stage to calculate Individual YLDs ^[3]^. We assumed a treatment duration of 1 month and untreated cases died or cured without treatment within 3 years as reported before ^[4]^.

Second, overall DALY before and during Ebola period were estimated by weighting sub-groups average DALY to the number of cases of the corresponding periods. The number of HAT cases reported was 154 and 59 before and during Ebola respectively. The number of reported cases over the 23 months before Ebola period was consistent with the previous WHO reports ^[5]^. We then assumed that 95 HAT cases, the difference between the cases reported before and during Ebola, were under-reported during Ebola period. The average DALY before Ebola was multiplied by the corresponding 154 HAT cases before Ebola to obtain overall DAYL before Ebola. The overall DAYL during Ebola period add up average DALY in reported cases during Ebola multiplied by 59 and average DALY in under-reported cases multiplied by 95.

Third, we randomly selected 1000 samples from our ‘HAT treatment’ database by bootstrapping to generate confidence intervals around our estimates.

1. Overall DALY = ∑ Average (YLL (i, g) + YLD (i, g)) * Number (g)

i: individual (1 to 213)

g: sub-groups (1 = reported cases before Ebola; 2 = reported cases during Ebola; 3 = under-reported cases during Ebola)

1. WHO | Metrics: Disability-Adjusted Life Year (DALY) [Internet]. WHO [cité 2017 juin 15];Available from: http://www.who.int/healthinfo/global_burden_disease/metrics_daly/en/

2. Robays J, Raguenaud ME, Josenando T, Boelaert M. Eflornithine is a cost-effective alternative to melarsoprol for the treatment of second-stage human West African trypanosomiasis in Caxito, Angola. Trop Med Int Health 2008;13(2):265‑71.

3. Fèvre EM, Odiit M, Coleman PG, Woolhouse ME, Welburn SC. Estimating the burden of rhodesiense sleeping sickness during an outbreak in Serere, eastern Uganda. BMC Public Health 2008;8:96.

4. Organization WH, WHO Expert Committee on the Control and Surveillance of Human African Trypanosomiasis (2013: Geneva S. Control and surveillance of human African trypanosomiasis: report of a WHO expert committee [Internet]. World Health Organization; 2013 [cité 2017 juin 15]. Available from: http://www.who.int/iris/handle/10665/95732

5. GHO | By category | Number of new reported cases (T.b. gambiense) - Data by country [Internet]. WHO [cité 2017 juin 15];Available from: http://apps.who.int/gho/data/node.main.A1636?lang=en
