# Supplementary material for: Impact of the Ebola outbreak on Trypanosoma brucei gambiense infection medical activities in coastal Guinea, 2014-2015: A retrospective analysis from the Guinean national Human African Trypanosomiasis control program
Source: PLoS Negl Trop Dis. 2017 Nov 13;11(11):e0006060. doi: 10.1371/journal.pntd.0006060 (PMC5703571; doi:10.1371/journal.pntd.0006060)
Supplement: S1 Table — (DOCX) [file pntd.0006060.s002.docx]

**S1 Table. Mean number of HAT patients treated before and during Ebola outbreak, Guinea (January 2012 to October 2015).**

|  | **N** | **Period length**  **(Months)** | **Mean (95% CI)**  **(Months)** |
| --- | --- | --- | --- |
| **Overall** | 213 | 45 | 5 (3 – 7) |
| Before Ebola | 154 | 23 | 7 (3 – 11) |
| During Ebola | 59 | 22 | 3 (2 – 3) |
