# Supplementary material for: Impact of the Ebola outbreak on Trypanosoma brucei gambiense infection medical activities in coastal Guinea, 2014-2015: A retrospective analysis from the Guinean national Human African Trypanosomiasis control program
Source: PLoS Negl Trop Dis. 2017 Nov 13;11(11):e0006060. doi: 10.1371/journal.pntd.0006060 (PMC5703571; doi:10.1371/journal.pntd.0006060)
Supplement: S2 Table — (DOCX) [file pntd.0006060.s003.docx]

**S2 Table. Characteristics of patients treated for HAT before and during Ebola outbreak, Guinea (January 2012 to October 2015),** *Cutoff December 2013*

|  | **N** | **Both**  **periods** | **Before Ebola outbreak**  **(22 months)** | **During Ebola outbreak**  **(23 months)** | *P value* |
| --- | --- | --- | --- | --- | --- |
| **Number of patients initiating the treatment** | 213 | 213 (100%) | 144 (68%) | 69 (32%) | -- |
| **Gender** | 212 |  |  |  | 0.6931 |
| Male |  | 130 (61%) | 89 (62%) | 41 (59%) |  |
| Female |  | 82 (39%) | 54 (38%) | 28 (41%) |  |
| **Age (years), n (%)** | 212 |  |  |  | **0.0236** |
| < 18 yrs |  | 51 (24%) | 41 (29%) | 10 (14%) |  |
| ≥ 18 yrs |  | 161 (76%) | 102 (71%) | 59 (86%) |  |
| **HAT treatment centers ^(1)^, n (%)** | 213 |  |  |  | **0.0195** |
| Boffa |  | 48 (23%) | 38 (26%) | 10 (15%) |  |
| Dubreka |  | 143 (67%) | 96 (67%) | 47 (68%) |  |
| Forecariah |  | 22 (10%) | 10 (7%) | 12 (17%) |  |
| **Occupation ^(2)^, n (%)** | 201 |  |  |  | 0.8084 |
| Outside rural activity |  | 73 (36%) | 51 (37%) | 22 (34%) |  |
| Outside city activity |  | 13 (**7%**) | 8 (6%) | 5 (8%) |  |
| Inside activity |  | 115 (57%) | 78 (57%) | 37 (58%) |  |
| **Type of screening, n (%)** | 211 |  |  |  | **<0.0001** |
| Passive screening |  | 107 (51%) | 48 (34%) | 59 (87%) |  |
| Active screening |  | 104 (49%) | 95 (66%) | 9 (13%) |  |
| **Disease clinic stage** ^(3)^**, n (%)** | 213 |  |  |  | **0.0121** |
| Phase 1 |  | 31 (15%) | 27 (19%) | 4 (6%) |  |
| Phase 2 |  | 182 (85 %) | 117 (81%) | 65 (94%) |  |
| **Treatment Status, n (%)** | 213 |  |  |  | **0.0015** |
| Treatment completed |  | 179 (84%) | 130 (90%) | 49 (71%) |  |
| Treatment not completed ^(4)^ |  | 32 (15%) | 13 (9%) | 19 (27%) |  |
| Dead |  | 2 (1%) | 1 (1%) | 1 (2%) |  |
| **Follow-up at 3 months, n (%)** | 213 |  |  |  | **<0.0001** |
| No |  | 139 (65%) | 78 (54%) | 61 (88%) |  |
| Yes |  | 74 (35%) | 66 (46%) | 8 (12%) |  |
| **Follow-up at 6 months, n (%)** | 213 |  |  |  | **0.0032** |
| No |  | 187 (88%) | 120 (83%) | 67 (97%) |  |
| Yes |  | 26 (12%) | 24 (17%) | 2 (3%) |  |
