# Supplementary material for: Impact of the Ebola outbreak on Trypanosoma brucei gambiense infection medical activities in coastal Guinea, 2014-2015: A retrospective analysis from the Guinean national Human African Trypanosomiasis control program
Source: PLoS Negl Trop Dis. 2017 Nov 13;11(11):e0006060. doi: 10.1371/journal.pntd.0006060 (PMC5703571; doi:10.1371/journal.pntd.0006060)
Supplement: S3 Table — (DOCX) [file pntd.0006060.s004.docx]

**S3 Table. Characteristics of patients treated for HAT before and during Ebola outbreak, Guinea (June 2012 to October 2015),** *Cutoff March 2014*

|  | **N** | **Both**  **periods** | **Before Ebola outbreak**  **(20 months)** | **During Ebola outbreak**  **(20 months)** | *P value* |
| --- | --- | --- | --- | --- | --- |
| **Number of patients initiating the treatment** | 154 | 154 (100%) | 100 (65%) | 54 (35%) | -- |
| **Gender** | 153 |  |  |  | 0.7754 |
| Male |  | 93 (61%) | 61 (62%) | 32 (59%) |  |
| Female |  | 60 (39%) | 38 (38%) | 22 (41%) |  |
| **Age (years), n (%)** | 153 |  |  |  | **0.0229** |
| < 18 yrs |  | 36 (24%) | 29 (29%) | 7 (13%) |  |
| ≥ 18 yrs |  | 117 (76%) | 70 (71%) | 47 (87%) |  |
| **HAT treatment centers ^(1)^, n (%)** | 154 |  |  |  | 0.4049 |
| Boffa |  | 21 (14%) | 14 (14%) | 7 (13%) |  |
| Dubreka |  | 111 (72%) | 69 (69%) | 42 (78%) |  |
| Forecariah |  | 22 (14%) | 17 (17%) | 5 (9%) |  |
| **Occupation ^(2)^, n (%)** | 144 |  |  |  | 0.7841 |
| Outside rural activity |  | 49 (34%) | 30 (32%) | 19 (38%) |  |
| Outside city activity |  | 10 (7%) | 7 (7%) | 3 (6%) |  |
| Inside activity |  | 85 (59%) | 57 (61%) | 28 (56%) |  |
| **Type of screening, n (%)** | 152 |  |  |  | **<0.0001** |
| Passive screening |  | 97 (64%) | 44 (44%) | 53 (100%) |  |
| Active screening |  | 55 (36%) | 55 (56%) | 0 (00%) |  |
| **Disease clinic stage** ^(3)^**, n (%)** | 154 |  |  |  | **0.0022** |
| Phase 1 |  | 24 (16%) | 22 (22%) | 2 (4%) |  |
| Phase 2 |  | 130 (84 %) | 78 (78%) | 52 (96%) |  |
| **Treatment Status, n (%)** | 154 |  |  |  | 0.3142 |
| Treatment completed |  | 123 (80%) | 82 (82%) | 41 (76%) |  |
| Treatment not completed ^(4)^ |  | 30 (19%) | 18 (18%) | 12 (22%) |  |
| Dead |  | 1 (1%) | 0 (0%) | 1 (2%) |  |
| **Follow-up at 3 months, n (%)** | 154 |  |  |  | **<0.0001** |
| No |  | 105 (68%) | 55 (55%) | 50 (93%) |  |
| Yes |  | 49 (32%) | 45 (45%) | 4 (7%) |  |
| **Follow-up at 6 months, n (%)** | 154 |  |  |  | **0.0205** |
| No |  | 140 (91%) | 87 (87%) | 53 (98%) |  |
| Yes |  | 14 (9%) | 13 (13%) | 1 (2%) |  |
