# Supplementary material for: Impact of the Ebola outbreak on Trypanosoma brucei gambiense infection medical activities in coastal Guinea, 2014-2015: A retrospective analysis from the Guinean national Human African Trypanosomiasis control program
Source: PLoS Negl Trop Dis. 2017 Nov 13;11(11):e0006060. doi: 10.1371/journal.pntd.0006060 (PMC5703571; doi:10.1371/journal.pntd.0006060)
Supplement: S4 Table — (DOCX) [file pntd.0006060.s005.docx]

**S4 Table.** **Disability-Adjusted Life Years (DALY) estimates before and during Ebola outbreak, Conakry Guinea 2012-2015 (Extended results)**

|  | **Before Ebola outbreak** |  | **During Ebola outbreak** | | | | |
| --- | --- | --- | --- | --- | --- | --- | --- |
|  | Reported cases  (n=154) |  | Reported cases  (n=59) |  | Under-reported cases (n=95) ^(1)^ | | |
|  |  |  |  |  | 2%  Case Fatally Rate | 5%  Case Fatally Rate | 10%  Case Fatally Rate |
| **Number of deaths** | 1.03 (0.96 - 1.09) |  | 1.31 (1.23 - 1.39) |  | 1.86 (1.75 – 1.97) | 4.85 (4.68 – 5.02) | 9.37 (9.14 – 9.60) |
| **Years Lost due to Disability (YLD)** |  |  |  |  |  |  |  |
| Average per person | 0.02 (0.02 – 0.02) |  | 0.02 (0.02 – 0.02) |  | 1.05 (1.05 - 1.05) | 1.05 (1.05 - 1.05) | 1.05 (1.05 - 1.05) |
| Overall | 3.98 (3.98 – 3.99) |  | 1.62 (1.62 – 1.62) |  | 99.75 (99.75 - 99.75) | 99.75 (99.75 - 99.75) | 99.75 (99.75 - 99.75) |
| **Years of Life Lost (YLL)** |  |  |  |  |  |  |  |
| Average per person | 0.29 (0.27 – 0.30) |  | 0.69 (0.65 – 0.74) |  | 0.78 (0.74 – 0.83) | 2.01 (1.93 – 2.08) | 3.92 (3.81 – 4.02) |
| Overall | 44.74 (42.01 – 47.47) |  | 41.15 (38.62 – 43.67) |  | 74.94 (70.36 – 79.52) | 191.14 (184.16 – 198.11) | 372.55 (362.78 – 382.33) |
| **Disability-Adjusted Life Year (DALY)** |  |  |  |  |  |  |  |
| Average per person | 0.31 (0.29 – 0.33) |  | 0.72 (0.68 – 0.76) |  | 1.83 (1.79 – 1.88) | 3.06 (2.98 – 3.13) | 4.97 (4.86 – 5.07) |
| Overall | 48.73 (46.73 – 51.47) |  | 42.77 (40.25 – 45.29) |  | 174.69 (170.11 – 179.27) | 290.89 (283.91 – 297.86) | 472.30 (462.53 – 482.08) |
| **Overall DALY increase**  **(***During Ebola - Before Ebola***)** | **--** |  | **--** |  | **168.73 (162.73 – 174.73)** | **284.92 (277.08 - 292.76)** | **466.34 (455.72 – 476.96)** |

^(1)^ Under-reported cases

^(2)^ The number of deaths in under-reported HAT cases was generated from a binomial distribution and their confidence interval from 1,000 bootstrap simulations

^(3)^ Values are mean values (with lower and upper 95% confidence intervals) from 1,000 bootstrap simulation
