# Supplementary material for: Impact of the Ebola outbreak on Trypanosoma brucei gambiense infection medical activities in coastal Guinea, 2014-2015: A retrospective analysis from the Guinean national Human African Trypanosomiasis control program
Source: PLoS Negl Trop Dis. 2017 Nov 13;11(11):e0006060. doi: 10.1371/journal.pntd.0006060 (PMC5703571; doi:10.1371/journal.pntd.0006060)
Supplement: S5 Table — (DOCX) [file pntd.0006060.s006.docx]

**S5 Table. HAT disease clinic stage before and during Ebola outbreak in patients detected through passive testing, Guinea (2012 to 2015)**

|  | **N** | **Both**  **periods** | **Before Ebola outbreak** | **During Ebola outbreak** | *P value* |
| --- | --- | --- | --- | --- | --- |
| **Disease clinic stage** ^(3)^**, n (%)** | 107 |  |  |  | -- |
| Phase 1 |  | 23 (21%) | 21 (43%) | 2 (3%) |  |
| Phase 2 |  | 84 (79%) | 28 (57%) | 56 (97%) | <0.001 |
